# Genome sequence of *Mediterraneibacter faecis* SZCH001, isolated from an autistic child

**DOI:** 10.1128/mra.00701-25

**Published:** 2025-09-12

**Authors:** Zhongsheng Zhu, Jiaojie Guan, Xiuxiu Xie, Chuqing Zhang, Zili Liao, Ziyuan Li, Boxian Lin, Hu Chen, Zeling Zhuang, Shiyun Meng, Yigui Zou, Wenwen Li, Mingjing Luo, Dongling Dai

**Affiliations:** 1 International Medical Center, Endoscopy Center and Gastroenterology Department, Shenzhen Children's Hospital 85113, Shenzhen, Guangdong, China; 2 State Key Laboratory of Quantitative Synthetic Biology, Shenzhen Institute of Synthetic Biology, Shenzhen Institutes of Advanced Technology, Chinese Academy of Sciences 631992, Shenzhen, China; 3 Shenzhen Children’s Hospital, Southern University of Science and Technology 255310, Shenzhen, China; 4 The Chinese University of Hong Kong 407605, Shenzhen, China; 5 Shenzhen University Health Science Center 477382, Shenzhen, China; University of Maryland School of Medicine, Baltimore, Maryland, USA

**Keywords:** *Mediterraneibacter faecis*, genome, autism spectrum disorder, gut microbiome

## Abstract

Here, we report the draft genome sequence of *Mediterraneibacter faecis* SZCH001, isolated from a fecal sample of an autistic child. The bacterial genome was sequenced using Illumina technology on a Novaseq 6000 platform. The assembled genome of *M. faecis* comprises 3,423,666 base pairs with a G+C content of 40.27%.

## ANNOUNCEMENT

*Mediterraneibacter faecis* is a gram-positive, anaerobic bacterium that is also known as ‘*Ruminococcus faecis*’. Although it was found to be more prevalent in adults with autism spectrum disorder (ASD) (1), its genomic characteristics remain underexplored. Here, we present the genome sequence of *M. faecis*, isolated from the feces of an autistic child enrolled in a broader study investigating microbial diversity in pediatric ASD cohorts.

A fecal sample from an 8-year-old male diagnosed with ASD was collected in a sample tube with anaerobic preservation medium (0.05% L-cysteine hydrochloride and 20% glycerol in PBS) and stored at −80°C until processing. All bacterial culture work was performed in an anaerobic chamber (10% H_2_, 5% CO_2_, and 85% N_2_). The sample was inoculated onto GAM agar (Hopebio, China) and incubated at 37°C for 48 h. Pure colonies were obtained after three subcultures for 48 h, then subjected to MALDI-TOF MS analysis (autoflex maX, Bruker, USA). One single colony identified as *M. faecis* was subcultured onto a fresh GAM agar plate for another 48-hour incubation at 37°C. Genomic DNA from this strain was extracted with the Wizard Genomic DNA Purification Kit (Promega, USA). Asequencing library was prepared using the TruePrep Flexible DNA Library Prep Kit for Illumina (Vazyme, China) by transposase-based fragmentation and adapter ligation. Final product around 350 bp was size selected by VAHTS DNA Clean Beads (Vazyme, China) and sent out for sequencing.

Raw paired-end reads were quality-filtered using Fastp (version 0.23.4) (2), removing adapters, low-quality reads (Q <15), and short reads (<15 bp). Genome assembly was performed with Unicycler (version 0.5.0) (3), and the assembled genome’s quality was assessed using QUAST (version 5.2.0) (4). The completeness of the genome was assessed using CheckM (5), yielding a value of 99.12%. The genome of *M. faecis* SZCH001 comprises 3,423,666 bp with a G+C content of 40.27%, distributed across 84 contigs, and an N_50_ fragment size of 150,785 bp. The genome was sequenced using 2.54 million 150 bp paired-end reads, achieving a coverage depth of 111×. Annotation using NCBI Prokaryotic Genome Annotation Pipeline (PGAP, version 6.10) (6) identified 3,221 coding sequences, 55 tRNAs, 6 rRNAs, and four ncRNAs. Average nucleotide identity (ANI) analysis revealed that *M. faecis* SZCH001 shares 95.97% ANI with type strain *M. faecis* JCM 15917 (accession GCA_001312505.1), and 93.29% ANI with Mediterraneibacter sp934309345 (accession GCF_934309345.1). One plasmid, *Enterococcus faecium* DO plasmid 1, was detected using PlasmidFinder (version 2.1.6) (7). Phage analysis using PHASTEST (8) did not identify any prophage regions. Whole genome-based taxonomic analysis was conducted using the Genome Taxonomy Database associated taxonomic classification toolkit (GTDB-Tk, version 2.4.0, database version r220) (9) with the parameter—classify_wf for standardized taxonomic classification, which validated the SZCH001 isolate as *M. faecis*. Default parameters were used for all software unless otherwise specified.

The isolation of *M. faecis* SZCH001 from an autistic child and the subsequent genomic insights provide a new perspective on the microbial components that might influence neurodevelopmental processes. While causality remains unestablished, this resource enables comparative genomics across ASD/neurotypical cohorts and functional studies of identified genes.

## Data Availability

The draft genome sequence of *M. faecis* SZCH001 has been deposited at GenBank under the accession number JBNCMN000000000. The genomes have been deposited in the NCBI under BioProject PRJNA1250271 and BioSample SAMN47936687. The raw Illumina reads are available at the NCBI Sequence Read Archive (SRA) under the accession number SRR33208113.
